# Effects of a peer-led educational intervention based on the theory of planned behavior on alcohol use intention and behavior among secondary school students in Northeast Ethiopia

**DOI:** 10.1371/journal.pone.0345099

**Published:** 2026-03-20

**Authors:** Yitbarek Wasihun, Morankar Sudhakar Narayan, Eshetu Girma

**Affiliations:** 1 Department of Health, Behavior and Society, Faculty of Public Health, Institute of Health, Jimma University, Jimma, Ethiopia; 2 School of Public Health, College of Medicine and Health Sciences, Tertiary Care Campus, Wollo University, Dessie, Ethiopia; 3 African Population and Health Research Center, Nairobi, Kenya; University of Tartu, ESTONIA

## Abstract

**Background:**

Adolescent alcohol use is a growing public health concern in Ethiopia. Theory-driven, peer-led educational interventions may modify psychosocial determinants of drinking behavior. This study assessed the effectiveness of a peer-led educational intervention grounded in the Theory of Planned Behavior (TPB) in changing alcohol-related intentions and self-reported alcohol consumption among secondary school students in Dessie and Kombolcha town, Northeast Ethiopia, high adolescent school enrollment.

**Methods:**

A quasi-experimental pretest-post test control study with *class-level allocation* was conducted in four public secondary schools in Northeast Ethiopia between February and June 2021. A total of 1,496 students aged 15–24 years were assigned to intervention (n = 748) or control (n = 748) groups. Full randomization at the individual student level was not feasible due to the risk of contamination within classrooms, so intact classes were used as units of allocation. The intervention consisted of four 60-minute peer-led sessions targeting TPB constructs and knowledge. Outcomes were assessed at baseline and three months post-intervention using validated self-administered questionnaires. *Generalized estimating equations* (GEE) *accounting for* class-level clustering were used to estimate adjusted effects.

**Results:**

At three months, intervention students demonstrated significantly higher alcohol-related knowledge (β = 4.76, 95% CI: 4.27–5.25), lower behavioral intention to drink (β = −1.03, 95% CI: −1.30 to −0.76), and reduced self-reported current alcohol use (adjusted OR = 0.58, 95% CI: 0.44–0.72; p < 0.001) compared with controls. Significant improvements were also observed in attitudes, subjective norms, and perceived behavioral control.

**Conclusions:**

The TPB-based peer-led school intervention was associated with short-term improvements in alcohol-related knowledge, psychosocial determinants, and reductions in intention and self-reported alcohol use. These findings are limited by the three-month follow-up, self-reported outcomes, and urban-only study sites. Randomized controlled trials with longer follow-up across diverse settings are warranted to assess sustainability and causal effects.

## Introduction

Alcohol use among adolescents remains a significant global public health concern, contributing to injury, mental health disorders, substance dependence, risky sexual behaviors, and poor academic outcomes [1,2]. Worldwide, over one-quarter of adolescents aged 15–19 years report alcohol consumption [3]. In Ethiopia, alcohol use among adolescents is substantial. The prevalence among school-aged adolescents and young adults is estimated at 27%, with peer influence, parental drinking, and concurrent substance use as key determinants [4–6].

Schools provide an important platform for preventive interventions during adolescence. Peer-led strategies are particularly promising because they leverage social modeling, normative influence, and age-specific communication [7].

The Theory of Planned Behavior (TPB) posits that behavior is predicted by intention, which is influenced by attitudes, subjective norms, and perceived behavioral control. Interventions targeting these constructs may reduce alcohol use. While TPB-informed interventions have demonstrated effectiveness in high-income settings [8,9], evidence from sub-Saharan Africa remains limited.

This study focused on Dessie and Kombolcha towns, chosen as representative urban centers in Northeast Ethiopia with high school enrollment and diverse socio-demographic profiles.

### Objectives

This study evaluated the effectiveness of a peer-led educational intervention based on the Theory of Planned Behavior (TPB) in reducing alcohol use intentions and self-reported alcohol use among secondary school students in Dessie and Kombolcha towns, Northeast Ethiopia.

### Study hypotheses

Post-intervention, intervention students will report (i) more negative attitudes, (ii) stronger anti-alcohol subjective norms, (iii) higher perceived behavioral control, (iv) lower intention to consume alcohol, and (v) reduced alcohol use.

## Methods

### Study design and setting

A quasi-experimental pretest-posttest control design with class-level allocation was conducted from February to June 2021 in four public secondary schools in Dessie and Kombolcha towns. Class-level allocation was used because full randomization at the student level was not feasible**,** due to the high risk of contamination among students within the same class.

### Participant and sample size

Students aged 15–24 years enrolled in grades 9–12 were eligible. The sample size was calculated to detect a 10% absolute reduction in current alcohol use, assuming 80% power, α = 0.05, baseline prevalence of 36.8%, and an intra-cluster correlation coefficient (ICC) of 0.02. The required sample size was 1,498; 1,496 students completed follow-up.

### Allocation procedure

Four of six eligible public secondary schools were randomly selected. Intact classes were randomly assigned to intervention or control arms using a computer-generated sequence prepared by an independent researcher. This clustered allocation minimized contamination while maintaining logistical feasibility.

### Participant recruitment, randomization, and study timeline

Students in grades 9–12 were stratified by grade within each selected school. Intact classes were used as the unit of allocation and were randomly assigned to intervention or control groups using a computer-generated random sequence by an independent researcher. A total of 1,496 students were allocated to intervention (n = 748) and control (n = 748) arms.

**Fig 1:**
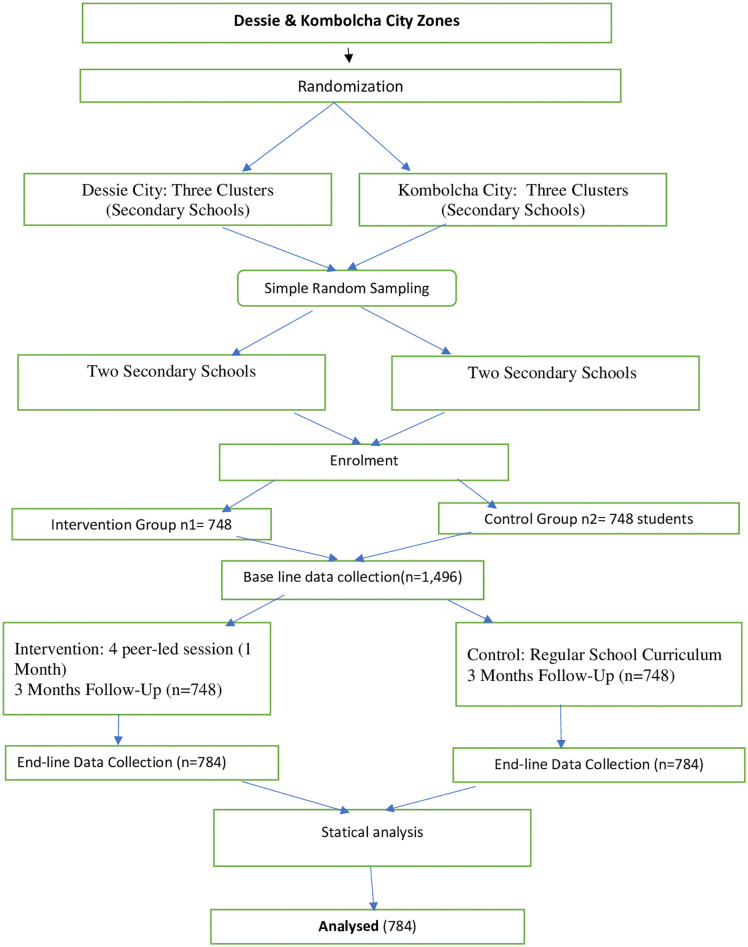
TREND diagram illustrating the flow of secondary school students through a quasi-experimental study aimed at reducing alcohol use intentions and behaviors in Dessie and Kombolcha, Northeast Ethiopia, 2021.

Baseline data were collected from 1–7 February 2021. The peer-led Theory of Planned Behavior–based intervention was delivered over four weeks (8 February–7 March 2021), followed by a three-month follow-up period. Post-intervention data were collected from 7–15 June 2021. Of 1,498 enrolled students, 1,496 completed follow-up (Fig 1).

### Intervention

The intervention consisted of four weekly 60-minute peer-led sessions targeting TPB constructs: knowledge, attitudes, subjective norms, perceived behavioral control, and refusal skills. Peer educators (n = 50) were trained in facilitation and TPB principles. The curriculum was systematically developed using: (1) an elicitation study to identify salient beliefs, (2) evidence from prior peer-led programs, and (3) expert review for cultural adaptation (S1 File).

### Outcome measures

#### Primary outcomes.

behavioral intention to consume alcohol and current alcohol use (past 30 days).

#### Secondary outcomes.

alcohol-related knowledge, attitudes, subjective norms, and perceived behavioral control.

Validated self-administered questionnaires adapted from TPB guidelines and prior studies were used [9–12]. Internal consistency was acceptable (α ≥ 0.77), and test-retest reliability was good (ICC = 0.81–0.93).

### Measurement TPB constructs and knowledge

**Behavioral intention to consume alcohol** was defined as a participant’s motivation or plan to drink within the next three months, regardless of current drinking status. It was measured using three items on a 7-point Likert scale (1 = very unlikely, 7 = very likely), summed to produce a total score ranging from 3 to 21, with higher scores indicating stronger intention (α = 0.994) [8,13].

**Attitudes toward alcohol consumption** was measured with four semantic differential items (range 4–28; α = 0.994) and corresponding belief-based evaluations (range –84 to +84; α = 0.864); higher scores denoted more favorable attitudes [8,13].

**Subjective norms** were measured with four 7-point Likert items (range 4–28; α = 0.992) and indirect measures of normative belief × motivation to comply (range –84 to +84; α = 0.937). Higher scores indicated positive perceived social approval [8,13].

**Perceived behavioral control (PBC)** was measured with four direct items (range 4–28; α = 0.890) and indirect control belief × perceived power items (range –105 to +105; α = 0.767); higher scores reflected greater perceived control [8,13].

**Alcohol-related knowledge** Alcohol-related knowledge was measured using 23 items on alcohol-related harms and common myths, with correct responses scored as one point each (range 0–23; α = 0.938), where higher scores indicated greater knowledge [12].

**Alcohol consumption status.** Never, ever, and current drinkers (past 30 days) based on NSDUH and WHO STEPwise surveillance definitions [14,15].

### Statistical analysis

Data were double-entered in EpiData 3.41 and analyzed in SPSS 25. Descriptive statistics summarized socio-demographics and outcomes. Normality was assessed via Shapiro–Wilk tests and Q–Q plots. Baseline group comparability was evaluated with chi-square and independent t-tests. Within-group changes: paired t-tests; between-group differences: independent t-tests.

Intervention effects were estimated using Generalized Estimating Equations (GEE) with class-level clustering (exchangeable correlation structure). Model fit: QIC. Missing data (<2%) handled via complete-case analysis. Statistical significance was defined as p < 0.05

### Ethical approval

The study was approved by the Institutional Review Board of Jimma University (Protocol No. JHRPGD/917/20). Written informed consent and assent were obtained. Confidentiality and voluntary participation were ensured.

## Results

### Baseline socio-demographic characteristics

A total of 1,496 students completed follow-up (748 intervention; 748 control). At baseline, groups were comparable in age, sex, and grade level. Differences were observed in parental education and family discussion about alcohol (p < 0.001). These variables were adjusted in multivariable analyses (Table 1).

**Table 1:** Sociodemographic characteristics of secondary school students in the intervention and control groups at baseline (N = 1,496).

Variable	Category	Intervention, n (%)	Control, n (%)	P-value
Sex	Male	377 (50.4)	360 (48.1)	0.408
	Female	371 (49.6)	388 (51.9)	
Age (years)	15–19	682 (91.2)	700 (93.6)	0.097
	20–24	66 (8.8)	48 (6.4)	
Grade level	Lower secondary (9–10)	321 (42.9)	318 (42.5)	0.917
	Upper secondary (11–12)	427 (57.1)	430 (57.5)	
Father’s educational status	No formal education	71 (9.5)	84 (11.2)	< 0.001*
	Primary education (1–8)	195 (26.1)	318 (42.5)	
	Secondary education (9–12)	142 (19.0)	216 (28.9)	
	Higher education (>12)	340 (45.5)	130 (17.4)	
Mother’s educational status	No formal education	100 (13.4)	118 (15.8)	< 0.001*
	Primary education (1–8)	283 (37.8)	495 (66.2)	
	Secondary education (9–12)	128 (17.1)	91 (12.2)	
	Higher education (>12)	237 (31.7)	44 (5.9)	
Discussion with parents about alcohol	Yes	186 (24.9)	129 (17.2)	< 0.001*
	No	562 (75.1)	616 (82.8)	

**Notes:** Values are n (%). P-values by chi-square test. *p < 0.05.

### Alcohol knowledge, TPB constructs, and alcohol use

At three-month follow-up, intervention students showed higher knowledge (Cohen’s d = 0.82), reduced favorable attitudes (d = −0.15), subjective norms (d = −0.15), increased perceived control (d = 0.13), decreased behavioral intention (d = −0.16), and reduced current alcohol use (OR = 0.56) at three months (Table 2).

**Table 2:** Comparison of intervention and control students at baseline and 3-month follow-up on alcohol-related knowledge, TPB constructs, and alcohol use (N = 1,496).

Outcome (Scale)	Group	Baseline, Mean ± SD/ n (%)	Follow-up, Mean ± SD/ n (%)	Within-group p-value	Between-group p-value	Effect Size (95% CI)
Knowledge (0–23)	Intervention	11.26 ± 6.89	16.18 ± 4.86	<0.001	<0.001	0.82 (0.74–0.90)
	Control	9.32 ± 7.69	9.49 ± 7.72	0.006	0.02	0.02 (−0.05–0.09)
Attitude, Direct (4–28)	Intervention	14.34 ± 7.93	13.20 ± 7.85	<0.001	<0.001	−0.15 (−0.23 to −0.07)
	Control	14.85 ± 5.94	14.92 ± 5.92	0.077	0.01	0.01 (−0.06–0.08)
Subjective Norms, Direct (4–28)	Intervention	14.11 ± 7.81	12.97 ± 7.71	<0.001	<0.001	−0.15 (−0.23 to −0.07)
	Control	14.79 ± 5.98	14.86 ± 5.96	0.077	0.01	0.01 (−0.06–0.08)
Perceived Behavioral Control, Direct (4–28)	Intervention	17.33 ± 5.76	18.11 ± 5.90	<0.001	<0.001	0.13 (0.05–0.21)
	Control	15.12 ± 3.59	15.10 ± 3.55	0.225	−0.01	(−0.08–0.06)
Attitude, Indirect (−84 to +84)	Intervention	61.90 ± 45.80	55.68 ± 44.51	<0.001	0.347	−0.14 (−0.22 to −0.06)
	Control	63.91 ± 32.71	64.17 ± 32.59	0.086	0.01	0.01 (−0.06–0.08)
Subjective Norms, Indirect (−84 to +84)	Intervention	47.36 ± 19.26	45.83 ± 18.72	0.001	0.005	−0.08 (−0.16 to 0.00)
	Control	48.66 ± 17.83	48.74 ± 17.78	0.132	0.00	0.00 (−0.07–0.07)
Perceived Behavioral Control, Indirect (−105 to +105)	Intervention	62.79 ± 27.81	63.17 ± 27.63	<0.001	0.982	0.01 (−0.07 to 0.09)
	Control	67.82 ± 25.59	68.05 ± 25.52	0.067	0.01	0.01 (−0.06–0.08)
Behavioral Intention (3–21)	Intervention	11.06 ± 5.97	10.09 ± 5.87	<0.001	<0.001	−0.16 (−0.24 to −0.08)
	Control	11.52 ± 4.85	11.58 ± 4.84	0.087	0.01	0.01 (−0.06–0.08)
Current Alcohol Use, n (%)	Intervention	173 (23.1)	108 (14.4)	<0.001	<0.001	OR=0.56 (0.43–0.72)
	Control	136 (18.2)	136 (18.2)	1.000	1.00	OR= (0.78–1.28)

**Notes:** Continuous data are mean ± SD; categorical data are n (%). Within-group: paired t-test/McNemar’s test; between-group: independent t-test/chi-square. Effect sizes: Cohen’s d (continuous), OR (categorical). All tests were two-sided, with *p* < 0.05 considered statistically significant.

### Adjusted intervention effects (GEE analysis)

At three months, intervention students had higher knowledge, greater perceived behavioral control, lower positive attitudes, lower subjective norms, and reduced behavioral intentions. Current alcohol use decreased in the intervention group (OR = 0.56; p < 0.001). Adjusted GEE analysis confirmed significant intervention effects (Table 3).

**Table 3:** Adjusted effects of a peer-led, Theory of Planned Behavior-based educational intervention on knowledge, psychosocial constructs, and alcohol use among secondary school students (N = 1,496).

Outcome	β/OR	SE	Wald	df	95% CI	P-value
Knowledge	4.76	0.25	361.76	1	4.27, 5.25	< 0.001
Attitude (Direct)	−1.21	0.18	45.11	1	−1.55, −0.86	< 0.001
Subjective Norms (Direct)	−1.21	0.18	44.89	1	−1.56, −0.87	< 0.001
Perceived Behavioral Control (Direct)	0.79	0.13	36.99	1	0.54, 1.05	< 0.001
Attitude (Indirect)	−6.48	1.03	39.56	1	−8.53, −4.43	< 0.001
Subjective Norms (Indirect)	−1.61	0.44	13.42	1	−2.48, −0.75	< 0.001
Perceived Behavioral Control (Indirect)	1.05	0.22	22.81	1	1.01, 1.72	< 0.001
Behavioral Intention	−1.03	0.14	54.32	1	−1.30, −0.76	< 0.001
Current Alcohol Consumption (Adjusted OR)	0.58	0.08	52.61	1	0.44, 0.72	< 0.001

**Notes:** β = GEE coefficient; OR = adjusted odds ratio. Adjusted for baseline knowledge, PBC, alcohol use, parental education, and family discussion. SE = standard error; 95% CI = confidence interval. Two-sided *p* < 0.05 was considered statistically significant.

## Discussion

This study found that a peer-led TPB-based intervention was associated with short-term improvements in knowledge and psychosocial determinants and with reductions in intention and self-reported alcohol use among secondary school students in Northeast Ethiopia. Baseline differences between intervention and control groups—including parental education, discussions about alcohol, baseline knowledge, perceived behavioral control, and alcohol use—were accounted for in adjusted Generalized Estimating Equations (GEE) analyses, strengthening confidence in the observed associations [15,16].

Students in the intervention group showed significant improvements in alcohol-related knowledge, whereas no such changes were observed in the control group**.** This observation is consistent with prior evidence that enhancing knowledge is a key component of effective preventive interventions [7,17].

The intervention was associated with changes in TPB constructs, including attitudes toward alcohol, perceived social norms, and perceived behavioral control. Reductions in favorable attitudes toward alcohol and perceived social approval for drinking suggest that the intervention may have influenced psychosocial factors that are central determinants of behavioral intention within the TPB framework [8,18]. Similar improvements in TPB constructs have been reported in other peer-led, theory-based interventions, among adolescents, where structured sessions strengthened attitudes, intentions, norms, and perceived control [4,16].

Behavioral intention to alcohol use decreased among intervention participants, accompanied by a reduction in self-reported alcohol use, whereas the control group showed minimal change. These findings are compatible with the TPB framework, in which changes in knowledge and psychosocial determinants are associated with behavioral outcomes**,** consistent with the predictive validity of TPB constructs [8,18].Comparable reductions in alcohol intentions and behaviors have been observed in peer-led interventions among adolescents and young adults, including BASICS programs [19,20].

Adjusted GEE models suggest that the observed associations persisted after controlling for baseline differences, indicating the potential value of structured, peer-facilitated, theory-driven interventions in school settings [15,16,20]. Collectively, these are consistent with prior literature suggesting that peer-led TPB-informed programs may influence short-term improvements in knowledge, psychosocial determinants, and alcohol-related behaviors among adolescents.

### Limitations

This study has several limitations that should be considered when interpreting the findings.

1. Alcohol use and psychosocial variables were self-reported, which may introduce recall and social desirability bias. The peer-led delivery could have amplified socially desirable responses. Future studies could incorporate objective or mixed-method measures to minimize reporting bias.
2. The quasi-experimental clustered design limits causal inference. Although analyses adjusted for baseline differences, residual confusion and potential contamination between groups cannot be excluded.
3. The three-month follow-up restricts conclusions regarding the sustainability of behavior change. Findings should be interpreted as short-term associations rather than long-term effects.
4. The study was conducted in only two urban towns, which may limit applicability to rural areas or other regions. These limitations are consistent with challenges reported in similar peer-led adolescent interventions [7,17,20]. Future research should consider cluster-randomized controlled trials with longer follow-up to confirm the durability and causal effects of peer-led AI-based interventions.

## Conclusion

The peer-led, Theory of Planned Behavior–based educational intervention was associated with improved alcohol-related knowledge, favorable changes in psychosocial determinants, lower behavioral intentions, and reduced self-reported alcohol consumption. Randomized controlled trials with longer follow-up are recommended.

## Supporting information

S1 File Educational intervention curriculum and session materials. (DOCX)

S2 File TREND Statement checklist. (DOCX)

S3 File Study protocol. (PDF)

## List of abbreviations:

TPB: Theory of Planned Behavior
PBC: Perceived Behavioral Control
SN: Subjective Norms
GEE: Generalized estimating equation
NSDUH: National Survey on Drug Use and Health
WHO STEPS: World Health Organization STEPwise approach to Surveillance
