# Supplementary material for: Effects of a peer-led educational intervention based on the theory of planned behavior on alcohol use intention and behavior among secondary school students in Northeast Ethiopia
Source: PLoS One. 2026 Mar 20;21(3):e0345099. doi: 10.1371/journal.pone.0345099 (PMC13004339; doi:10.1371/journal.pone.0345099)
Supplement: S1 File — (DOCX) [file pone.0345099.s001.docx]

Intervention Facilitator Guideline

**Peers for Change:**

A Peer-Led School-Based Intervention to Reduce Alcohol Use Intentions and Behaviors Among secondary school students in Dessie and Kombolcha, Northeast Ethiopia. Grounded in the Theory of Planned Behaviour (TPB): A Quasi-Experimental Intervention Model

## **1. Introduction**

### **Purpose of the Manual**

This manual is intended to guide trained peer facilitators delivering the Peers for Change intervention—a theory-driven, school-based prevention program designed to reduce the intention and likelihood of alcohol use among secondary school students. The guideline supports peer facilitators in delivering accurate knowledge, enhancing psychosocial skills, and promoting behaviour change strategies in students, grounded in the Theory of Planned Behaviour (TPB).

## **2. Overview of the Intervention**

Peers for Change is a primary prevention intervention focused on preventing the initiation and continuation of alcohol use among students. The intervention, structured around four weekly, peer-facilitated sessions, is implemented in the school setting during regular academic hours and targets students at all stages of use—non-users, ever-users, and current users.

### **Theoretical Framework:**

This intervention is based on the Theory of Planned Behaviour, which postulates that behaviour is shaped by 3 key constructs:

·       Attitudes and behavioural beliefs toward alcohol use (positive or negative beliefs about substance use)

·       Subjective norms and normative beliefs (social pressures and perceived expectations)

·       Perceived behavioural control and control beliefs (confidence in one’s ability to resist)

By targeting these three constructs, and knowledge, the program aims to reduce both behavioural intentions and actual alcohol use behaviours.

3. Aims of the Intervention

The intervention sought to:

- Promote unfavourable evaluations of alcohol use among students
- Strengthen students’ perceptions that use of alcohol is socially discouraged by peers, family, and teachers.
- Strengthen PBC and self-efficacy in resisting peer pressure and make independent, alcohol, -free choices.
- Improve knowledge about and risk perception about alcohol targeting the immediate and long-term health and social consequences due to alcohol use.
- Ultimately, reduce both behavioural intentions and actual alcohol use behaviour among students.

4. Intervention Delivery

The intervention was delivered through four weekly, in-group sessions, during regular school hours, each lasting approximately 60 minutes. A total of 25 peer groups were formed, with each group comprising 30 students from grades 9 to 12, randomly selected class students. Each session was co-facilitated by two trained peer educators—one the lead facilitator and another offering support. Peer educators received structured training on facilitation techniques, ethical considerations, and content delivery.

The session was organised around four thematic modules, targeting fundamental TPB constructs, including alcohol-related knowledge and risk perception, attitudes, subjective norms, and perceived behavioural control. All materials were developed using evidence-based educational frameworks and were delivered using participatory and experiential learning techniques.

Peer facilitators received structured training on ethical conduct, communication, and content delivery prior to implementation.

5. Post-Intervention Follow-Up

Three months after completion, a follow-up assessment evaluated the sustainability of the program’s outcomes. Outcome data were collected simultaneously from both the intervention and control schools using standardised assessment tools. Control schools did not receive any additional programming during the intervention period. However, educational materials were provided after the study concluded to ensure equitable benefit.

6. Session Structure

Each session was conducted according to a standardised delivery format detailed in the facilitator’s manual: This format included:

- Clear session objectives
- An overview of the session content
- A list of required materials
- Step-by-step procedures, each accompanied by estimated time allocations
- Participant feedback

7. Module Descriptions and Activities

Module 1: Knowledge and Risk Perception about Alcohol.

Duration: 60 minutes

Purpose:
It is designed to equip participants with essential knowledge about the detrimental effects of alcohol consumption. It intends to enhance the perceptions of the risks linked to alcohol, and correct common misconceptions.

Objectives:

- Participants understand both the immediate and long-term health risks related to the use of alcohol.
- Recognised and identified the harmful chemical constituents found in these substances
- Evaluate the benefits of abstaining from alcohol use.
- Develop and strengthen behavioural intentions to remain alcohol free.

Expected Healthy Behavioural Outcomes (HBOs):

- Reduction in the initiation of alcohol use
- Increased motivation to cease use among current or experimental alcohol users.
- Strengthened peer norms supporting alcohol-free lifestyles.

Key Activities:

1.     Interactive Presentation (35 min):

Peer educators deliver an engaging session on the health, psychological, and social consequences of alcohol use. The presentation also clarifies common misconceptions and misinformation using age-appropriate language to ensure understanding.
2. Small-Group Discussion and Reflections (20 minutes):

Students break into small groups to discuss and reflect on what they learnt during the interactive presentation. This activity encourages critical thinking and deep understandings of the session’s key messages. Participants are encouraged to:

- Relate the presented risks to real-life scenarios within their peer and community context
- Share thoughts on how alcohol use may affect personal goals and relationships
- Explore how knowledge influences their current or future intentions regarding use

This activity helps internalise the material through peer dialogue and personal storytelling, fostering deeper engagement.

3. Participant Feedback (5 minutes)
Students complete a short feedback form to assess:

- Their understanding of the session content
- The clarity and usefulness of the information
- Suggestions for improving the session or addressing unanswered questions

Facilitators collect feedback to evaluate learning outcomes and refine delivery in future sessions.

Resources Required:

·       Student handouts and leaflets

·       Feedback surveys

Module 2: Attitudes, Behavioural Beliefs, and Coping with Anxiety

Duration: 60 minutes

Purpose:
This intervention module is prepared to enhance negative attitudes and beliefs toward the use of alcohol by providing healthy alternatives—specifically, effective anxiety management techniques. The goal of this is to reduce emotional reliance on alcohol by providing students with practical coping mechanisms.

Objectives:

- Recognise how being alcohol free can positively influence personal health, academic performance, and future life goals.
- Build confidence and a sense of identity around living free from alcohol.
- Understand and practice anxiety and stress management techniques as healthier alternatives to using alcohol.

Key Activities:

Activity 1: Structured Debate (45 minutes)

Debate topic: Is using substances like alcohol good or bad for someone your age?

Purpose:
To encourage participants critical thinking by examining beliefs, attitudes, and the potential consequences of alcohol use among adolescents and youth, through collaborative debate and reflection, students are empowered to form well-informed opinions.

Procedure:

1.     Group Assignment (5 minutes):
To encourage critical thinking, students were divided into two balanced groups.

- - Group one: Assigned to argue in favour of the idea that using alcohol may have benefits for people their age.
  - Group two: Assigned to argue against using alcohol, indicating the risks and negative consequences. A lottery method was used to randomly determine which group selected their stance first.

This activity isn’t meant to promote anyone to use substances. instead, to help students explore deeply about commonly held beliefs, reflect about peer pressures they face, and build stronger personal convictions through open dialogue.

Note to Facilitators:
Before the debate begins, remind participants that this is a learning exercise, and they may be asked to argue a side they do not personally agree with—and that’s okay. The goal is to develop communication skills in argumentation, develop critical thinking, and gain a better understanding of different perspectives. Respectful discussion is key, even when opinions differ.

2.     Argument Development (15 minutes):
Groups worked in each group to explore arguments supporting their assigned viewpoint. During the activity, participants were motivated to:

- - Assessed the short- and long-term effects of using substances like alcohol.
  - Explore how decisions and behaviours related to health were shaped by individuals’ attitudes and beliefs.
  - Discuss the other risks associated with alcohol use behaviours.
  - Discuss the influence of school and community environments on alcohol norms.

3.     Debate Preparation (5 minutes):
Three representatives of each group were selected to present their arguments. The selected representatives work with their group members to collect ideas and prepare their key points for the debate.

4.     Debate Session (10 minutes):
The session was facilitated by a peer leader; each group presented its key arguments, followed by brief rebuttals. Respectful dialogue and critical analysis were encouraged throughout the session.

5.     Reflection and Note-Taking (10 minutes):
After the debate, students took time to reflect individually on what they had learnt and jot down their personal thoughts and takeaways. The peer facilitator guided this process to help consolidate insights and encourage deeper understanding of the topic.

### **Activity 2:** Relaxation Techniques (15 min):

### **Techniques for Coping with Anxiety (Based on Botvin, 1980)**

Purpose:
To introduce participants to effective tension-reduction methods as healthy alternatives to using alcohol for managing stress and anxiety.

Procedure:

At the start, students were informed that many of their peers use alcohol as coping mechanisms for stress and anxiety. To provide healthier coping strategies, students were engaged in tension-reduction exercises, including relaxation techniques and deep breathing. Students were encouraged to practice these methods, with an emphasis on how relaxation fosters calmness and naturally alleviates anxiety.

- Relaxation Exercise (5 minutes):
  Students engaged in a guided relaxation practice, consisting of the following steps:

1.     Sitting comfortably and quietly.

2.     Closing their eyes gently.

3.     Slowly relaxing muscles throughout the body, beginning at the toes and moving upward to the head and face.

4.     Playing close attention to their breathing, inhaling relaxation, and exhaling tension with each breath.

5.     Visualising a serene and peaceful place, allowing themselves to feel completely relaxed and at ease.

To support the experience, soothing music was played during the exercise.

Homework:
Students were instructed to practice this relaxation exercise once daily, either at home or on the school campus.

- Deep Breathing Exercise (5 minutes):
  Students were led through a deep breathing technique designed to reduce physical tension linked to anxiety:

1.     Slowly inhaling through the nose, filling the diaphragm with air (without moving the chest) for a count of four.

2.     Exhaling slowly and completely for a count of eight.

3.     Repeating this breathing cycle four to five times to promote relaxation

### **Activity 3: Participant Feedback (5 minutes)**

At the end of the session, students completed feedback surveys and submitted them before leaving.

Module 3: Subjective Norms/Normative Beliefs and Decision-Making

Duration: 60 minutes

Purpose:
This module examines how social influences—family, friends, teachers, and classmates—impact decisions on alcohol use. It intends to strengthen students’ decision-making abilities and make informed, healthy choices by understanding these social dynamics.

Objectives:
Aims to empower students to:

- Recognise the influence of family members, teachers, friends, and classmates on their decisions to use alcohol.
- Enhance problem-solving and decision-making skills to handle substance use situations.
- Develop practical coping strategies to face challenges such as parental alcohol, use and peer pressure in a resilient way.

Key Activities:

Activity 1: Discussion: Approvers vs. Disapprovers of alcohol (40 min):

Instructions:

Facilitate a guided group reflection on who in their lives approves or disapproves of alcohol use: Use the following questions to prompt thoughtful discussion about favourable social interactions and influences.

- - What positive things do you do for your family members, and what positive things do they do for you?
  - What positive things do you do for your friends, and what positive things do they do for you?
  - What positive things do you do for your teachers, and what positive things do they do for you?
  - What actions have you taken to help family members or friends to reduce their use of alcohol?

Encourage students to consider how these relationships can shape their choices around alcohol use—both in supportive and challenging ways.

Use the following questions to guide and deepen the group discussion.

| Important/significant others | Would they approve or  disapprove of your  alcohol use behavior? | How would they  influence your  alcohol use behavior? | How would you  influence them  alcohol use behavior? |
| --- | --- | --- | --- |
| Parents | 1.Approve 2. Disapprove |  |  |
| Other family members | 1.Approve 2. Disapprove |  |  |
| Close friends | 1.Approve 2. Disapprove |  |  |
| Teachers | 1.Approve 2. Disapprove |  |  |

### **Activity 2: Decision-Making Practice (Based on Botvin, 1980) (15 minutes)**

Purpose:
This is to provide students with informed decisions—especially when facing challenges about alcohol use.

Procedure: Using these steps:

1.     Clarify the decision or problem:
Support participants in identifying the issue that needs to be solved.
Example question: What is the problem you are facing with alcohol use?

2.     Consider possible choices:
Support participants to brainstorm various options or actions they could take to tackle the issue.
Example question: What are the different choices or options you have?

3.     Gather additional information if needed:
Support participants to identify if more facts or details are necessary to make a good decision; if not, what information they still need.
Example question: Do you have enough information to decide? If not, what else do you need to find out?

4.     Evaluate consequences:
Support participants to evaluate the advantage and disadvantage of each option by considering the potential outcomes of their choices.

          Example question: What are the advantages and disadvantages of each option?

5.     Make and follow through with the decision:
Support participants to select the best option based on their evaluation and commit to taking action.

Example prompt: “Which choice will you make, and how will you follow through with it?”

### **Activity 3: Participant Feedback (5 minutes)**

At the end of the session, students completed feedback surveys and submitted them before leaving.

Module 4: Perceived Behavioural Control and Self-Efficacy

Duration: 60 minutes

Purpose:

This is to develop practical skills and confidence in resisting peer pressure and managing situations of alcohol use.

Objectives:

- To set clear and realistic personal goals to stay free from substances, particularly alcohol.
- To analyse potential barriers to reaching these goals and develop effective mitigation strategies.
- To enable students to say no in peer and social situations in culturally appropriate ways.

Key Activities:

### **Activity 1: G**oal-Setting and Barrier Analysis (30 min):

### **Purpose:** To encourage students to reflect on their personal control over alcohol-related behaviour and help them set clear, actionable goals to remain substance-free.

Procedure:

·       Students were divided into small groups for focused discussion using the following guiding questions:

o   What have you done to control alcohol use—either in your own life or by supporting your friends or family?

o   What are your personal goals for avoiding alcohol? (Students were encouraged to make these goals specific and realistic.)

o   What barriers might make it difficult to achieve these goals, and how could you overcome them?

·       After the group discussions, a few students were invited to share their insights, control strategies, and goals with the larger group.

·       The facilitator recorded key ideas and strategies using the table below. Students who identified as non-users were asked to consider what factors might put them at risk for alcohol use in the future. And what steps can you take to manage those risks?

| Behavior | Goals | Barriers | Strategies to overcome |
| --- | --- | --- | --- |
| Own alcohol use |  |  |  |
| Friends’ alcohol use |  |  |  |
| Parents’ alcohol use |  |  |  |

### **Activity 2: Refusal Skill – Resisting Peer Pressure (25 minutes)**

Purpose:
To help students identify situations involving peer pressure and practice assertive communication skills for refusing offers of alcohol.

Procedure:

1.     Identifying Peer Pressure Scenarios (10 minutes):

·       Students were invited to describe real or imagined situations they had seen, heard about or experienced someone was pressured by peers to use alcohol.

·       Volunteers shared examples, and the facilitator listed these peer-pressure scenarios on the blackboard.

·       The group briefly discussed each scenario, focusing on the social dynamics, risks involved, and challenges in those situations.

2.     Role Play: Resisting Peer Pressure (15 min):

- Students were divided into pairs or small groups. Each group selected one of the listed scenarios to role-play.
- Each student had the opportunity to practice using assertive communication to refuse an offer of alcohol.
- Facilitators provided feedback and encouraged students to reflect on what strategies felt most natural and effective.
- Key skills emphasized included:
- Saying “no” clearly and confidently
- Using body language and tone that matches their message
- Offering alternative activities or responses
- Removing themselves from the situation if needed
- The facilitator and peers provided supportive feedback after each role play.

Activity 3. Participant Feedback (5 min): Session evaluation.

Summary of Facilitator Protocol

| **Module** | **TPB Constructs & knowledge Targeted** | **Key Activities** | **Total Time**  **(Minutes)** |
| --- | --- | --- | --- |
| Module 1 | Knowledge & Risk Perception | Interactive session, reflections,  Feedback | 60 |
| Module 2 | Attitudes, behavioral & Coping Skills | Structured debate, relaxation  training, feedback | 60 |
| Module 3 | Subjective Norms, normative beliefs & Decision-Making | Discussions, decision-making  practice, feedback | 60 |
| Module 4 | PBC, Self-Efficacy & Refusal Skills | Goal-setting, role-play,  strategy sharing | 60 |

**8. Monitoring and Fidelity Assurance**

To maintain the consistency of the intervention, peer educators were supervised by trained school staff and members of the research team to ensure fidelity to the intervention protocol. Implementation fidelity was assessed using structured observation checklists, session logs, and participant feedback surveys. Additionally, baseline and follow-up data were collected using validated instruments. All data collection procedures adhered to ethical standards and data quality assurance protocols.

### **9. Ethical and Cultural Considerations**

To ensure a respectful and effective learning environment, the following ethical and cultural principles were upheld:

- Participation was entirely voluntary, with full respect for students' privacy and confidentiality.
- Sessions were conducted in a safe, inclusive, and non- judgmental atmosphere.
- All content, examples, and language were tailored to be age-appropriate and culturally relevant to the students’ context.
