# Supplementary material for: Effects of a peer-led educational intervention based on the theory of planned behavior on alcohol use intention and behavior among secondary school students in Northeast Ethiopia
Source: PLoS One. 2026 Mar 20;21(3):e0345099. doi: 10.1371/journal.pone.0345099 (PMC13004339; doi:10.1371/journal.pone.0345099)
Supplement: S3 File — (PDF) [file pone.0345099.s003.pdf]

STUDY PROTOCOL

# Effects of a Peer-Led Educational Intervention Based on the Theory of Planned Behavior on Alcohol Use Intention and Behavior Among Secondary School Students in Northeast Ethiopia

**Protocol Version:** 1.0 (January 15, 2021)

**Ethics Committee Approval:** Protocol No. JHRPGD/917/20 (Jimma University Institutional Review Board)

**Authors:**

Yitbarek Wasihun[1,2]*, Morankar Sudhakar Narayan[1], Eshetu Girma[3]

**Affiliations:**

[1]Department of Health, Behavior and Society, Faculty of Public Health, Institute of Health, Jimma University, Jimma, Ethiopia

[2]School of Public Health, College of Medicine and Health Sciences, Tertiary Care Campus, Wollo University, Dessie, Ethiopia

[3]African Population and Health Research Center, Nairobi, Kenya

***Corresponding author:**

Yitbarek Wasihun

Department of Health, Behavior and Society

Faculty of Public Health, Institute of Health

Jimma University

Jimma, Ethiopia

Email: yitbarek2003@gmail.com

## *Abstract*

***Background:*** Adolescent alcohol use is a growing public health concern in Ethiopia. Theory-driven, peer-led educational interventions may modify psychosocial determinants of drinking behavior. This protocol describes a study designed to assess the effectiveness of a peer-led educational intervention grounded in the Theory of Planned Behavior (TPB) in changing alcohol-related intentions and self-reported alcohol consumption among secondary school students in Dessie and Kombolcha towns, Northeast Ethiopia.

***Methods:*** A quasi-experimental pretest-posttest control group design with class-level allocation will be conducted from February to June 2021 in four public secondary schools in Northeast Ethiopia. A total of 1,496 students aged 15–24 years will be assigned to intervention (n = 748) or control (n = 748) groups. Full randomization at the individual student level is not feasible due to the risk of contamination within classrooms, so intact classes will be used as units of allocation. The intervention consists of four weekly 60-minute peer-led sessions targeting TPB constructs and alcohol-related knowledge. Outcomes will be assessed at baseline and three months post-intervention using validated self-administered questionnaires. Primary outcomes are behavioral intention to consume alcohol and current alcohol use (past 30 days). Secondary outcomes include alcohol-related knowledge, attitudes, subjective norms, and perceived behavioral control. Generalized estimating equations (GEE) accounting for class-level clustering will be used to estimate adjusted effects.

***Discussion:*** This study will provide evidence on the effectiveness of a culturally adapted, peer-led, theory-based intervention to reduce alcohol use intentions and behaviors among secondary school students in Northeast Ethiopia. Findings may inform school-based prevention programs and policies in similar settings.

***Keywords:*** Alcohol use; adolescents; peer-led intervention; Theory of Planned Behavior; Ethiopia; school-based prevention; study protocol

# *BACKGROUND*

## *Global Burden of Adolescent Alcohol Use*

Alcohol consumption among adolescents represents a critical public health challenge worldwide. According to the World Health Organization's Global Status Report on Alcohol and Health (2018), approximately 26.5% of all adolescents aged 15-19 years globally are current drinkers [1].The harmful use of alcohol during adolescence contributes to approximately 320,000 deaths annually among young people aged 15-29 years [2].

Adolescent alcohol use is associated with numerous adverse outcomes, including increased risk of injuries and accidents [3]; impaired brain development and cognitive function [4]; mental health disorders, including depression and anxiety [5]; risky sexual behaviors leading to unintended pregnancies and sexually transmitted infections [6]; poor academic performance and school dropout [7]; and increased likelihood of developing alcohol use disorders in adulthood [8].

## *Alcohol Use in Ethiopia*

In Ethiopia, adolescent and youth alcohol use has emerged as a growing public health concern. Nationally representative data from the 2016 Ethiopian Demographic and Health Survey (EDHS) documented that among youth aged 15-24 years, 36.8% had consumed alcohol in the preceding month, 12.6% had chewed khat, and 0.95% had used tobacco [9]. These findings highlight the substantial burden of substance use among young Ethiopians and underscore the urgent need for effective prevention interventions.

A systematic review and meta-analysis by Ali and Worku (2020) estimated the prevalence of current alcohol consumption among school adolescents and youths in Ethiopia at 27%, with significant regional variation [10]. Regional studies have reported varying prevalence rates: Gondar preparatory schools at 31.2% [11], Nekemte secondary schools at 24.6% [12] , Addis Ababa high schools at 28.3% [13], and Jimma secondary schools at 26.8% [14].

Key determinants of adolescent alcohol use in Ethiopia include peer pressure, parental drinking behavior, accessibility of alcohol, low perceived risk, and poor knowledge of alcohol-related harms [10–14] . The EDHS data further reveal that alcohol use among Ethiopian youth is more prevalent in urban areas and among those with lower educational attainment, highlighting the vulnerability of in-school and out-of-school youth populations [9].

## *Theoretical Framework: Theory of Planned Behavior*

The Theory of Planned Behavior (TPB), developed by Icek Ajzen (1991), provides a robust framework for understanding and predicting health behaviors [15]. According to TPB, behavioral intention is the immediate antecedent of behavior, and intention is determined by three independent constructs:

1. **Attitude toward the behavior:** The degree to which a person has a favorable or unfavorable evaluation of the behavior
2. **Subjective norm:** The perceived social pressure to perform or not perform the behavior
3. **Perceived behavioral control:** The perceived ease or difficulty of performing the behavior, which also has a direct effect on behavior

TPB has been successfully applied in numerous studies predicting and changing alcohol-related behaviors among adolescents [16–18]. Interventions based on TPB have demonstrated effectiveness in reducing alcohol consumption intentions and behaviors in high-income countries [19, 20].

## *Peer-led Educational Interventions*

Peer-led interventions leverage social influence processes that are particularly relevant during adolescence. Systematic reviews have shown that peer-led approaches can be effective in reducing substance use among young people [21, 22]. The advantages of peer-led interventions include enhanced credibility and relatability of messages, utilization of existing social networks, normalization of health-promoting behaviors, cost-effectiveness and sustainability, and empowerment of peer educators.

## *Rationale for the Study*

Despite the growing burden of adolescent alcohol use in Ethiopia, as evidenced by national surveys showing that over one-third of youth consume alcohol [9], and the documented effectiveness of theory-based peer-led interventions in other settings, there is limited evidence from sub-Saharan Africa, particularly Ethiopia. Most existing studies in Ethiopia have focused on prevalence and associated factors, with few intervention studies [23, 24].

The Northeast Ethiopia region, specifically Dessie and Kombolcha towns, was selected because of high secondary school enrolment rates providing access to a large adolescent population; diverse socio-demographic characteristics representative of urban Ethiopia; limited

existing alcohol prevention programmes in schools; and reports of increasing alcohol availability and consumption among youth.

This study aims to address the evidence gap by developing and evaluating a culturally adapted, peer-led educational intervention grounded in the Theory of Planned Behavior to reduce alcohol use intentions and behaviors among secondary school students.

## Study Objectives

This study aims to evaluate the effectiveness of a peer-led educational intervention based on the Theory of Planned Behavior (TPB) in reducing alcohol use intentions and self-reported alcohol use among secondary school students in Dessie and Kombolcha towns, Northeast Ethiopia.

## Study Hypotheses

Post-intervention, intervention students will report:

1. More negative attitudes toward alcohol use
2. Stronger anti-alcohol subjective norms
3. Higher perceived behavioral control to resist alcohol
4. Lower intention to consume alcohol
5. Reduced self-reported alcohol use

# METHODS

## Study Design and Setting

A quasi-experimental pretest-posttest control group design with class-level allocation will be conducted from February to June 2021 in four public secondary schools in Dessie and Kombolcha towns, Northeast Ethiopia. This design was selected because individual-level randomization was not feasible due to the high risk of contamination within classrooms. Class-level allocation preserves the natural classroom environment, minimizes diffusion of intervention effects, and maintains logistical feasibility.

Dessie and Kombolcha towns are located in the South Wollo Zone of the Amhara National Regional State, approximately 400 kilometers northeast of Addis Ababa. The towns have a combined population of approximately 350,000 and serve as commercial and educational hubs

for the surrounding districts. The study will include public secondary schools offering grades 9-12.

This study protocol and the subsequent full report will adhere to the Transparent Reporting of Evaluations with Nonrandomized Designs (TREND) statement. A completed TREND checklist will be submitted as a supplementary file (S1 File) upon manuscript submission to ensure comprehensive and transparent reporting of all study elements,

**Dates of Recruitment**

| Phase | Duration | Dates |
|---|---|---|
| Baseline data collection | 1 week | February 1-7, 2021 |
| Intervention delivery | 4 weeks | February 8 - March 7, 2021 |
| Follow-up period | 3 months | March 8 - June 6, 2021 |
| Endline data collection | 1 week | June 7-15, 2021 |

## *Participant Characteristics*

**Source Population:** All secondary school students enrolled in grades 9-12 in public secondary schools in Dessie and Kombolcha towns during the 2020/2021 academic year.

**Study Population:** Students aged 15-24 years enrolled in selected secondary schools who meet the eligibility criteria.

**Inclusion and Exclusion Criteria**

| Inclusion Criteria | Exclusion Criteria |
|---|---|
| Currently enrolled in grades 9-12 in selected public secondary schools | Unable to comprehend and complete the questionnaire due to cognitive or language barriers |

| Inclusion Criteria | Exclusion Criteria |
|---|---|
| Aged 15-24 years at the time of enrolment | Planning to transfer to another school during the study period |
| Provide written informed consent (if ≥18 years) or parental consent and child assent (if 15-17 years). | Currently participating in another alcohol prevention programme |
| Available for the duration of the study (February to June 2021) | Severe mental or physical illness that would preclude participation |

## *Sample Size Determination*

The sample size is calculated using the formula for comparing two proportions in cluster-based studies [24], accounting for the intra-cluster correlation coefficient (ICC). Assuming 80% power, 95% confidence level ($\alpha = 0.05$), baseline prevalence of current alcohol use of 36.8% **[9]**, and expected post-intervention prevalence of 26.8% (representing a 10% absolute reduction), the required sample size was determined. To account for clustering at the class level, an ICC of 0.02 was applied based on similar school-based studies [25], with an average cluster size of 30 students per class, yielding a design effect of 2. After adjusting for a 10% loss to follow-up, the total required sample was 1,496 students (748 per group).

## *Sampling and Allocation Procedure*

**School Selection:** From the six eligible public secondary schools in Dessie and Kombolcha towns, four schools will be randomly selected using a computer-generated random number sequence. Two schools will be selected from Dessie and two from Kombolcha to ensure geographic representation.

**Class Selection and Allocation:** Within each selected school, classes in grades 9-12 will be stratified by grade level. From each grade, classes will be randomly assigned to either intervention or control arms using a computer-generated random sequence prepared by an independent researcher not involved in data collection. The allocation sequence will be generated prior to recruitment, and allocation concealment will be maintained by using

sequentially numbered, opaque, sealed envelopes to prevent selection bias. All students in selected classes will be invited to participate.

**Allocation Ratio:** 1:1 (intervention: control)

**Rationale for Class-Level Allocation:** This approach minimizes contamination between intervention and control participants, maintains logistical feasibility, and preserves the natural classroom environment.

**DETAILED DESCRIPTION OF THE PROTOCOLS/INTERVENTIONS UTILIZED**

**Intervention Overview:**

| Feature | Description |
|---|---|
| Type | Primary prevention |
| Duration | Four weekly 60-minute sessions |
| Setting | School classrooms during regular hours |
| Facilitators | Trained peer educators (2 per group, total 50) |
| Group size | 30 students per group (25 groups) |

## *Theoretical Foundation*

The intervention will be systematically developed based on the Theory of Planned Behavior, targeting each of the TPB constructs:

| TPB Construct | Intervention Target | Activities |
|---|---|---|
| Attitude | Reduce favorable attitudes toward alcohol | Discuss negative consequences, share personal stories, examine media messages |
| Subjective Norms | Correct misperceptions about peer alcohol use | Present actual prevalence data, discuss social pressures, and role-play resistance. |

| TPB Construct | Intervention Target | Activities |
|---|---|---|
| Perceived Behavioral Control | Enhance self-efficacy to refuse alcohol | Practice refusal skills, develop coping strategies, build confidence |
| Intention | Reduce intention to consume alcohol | Set personal goals, make public commitments, develop action plans |

## *Curriculum Development Process*

The intervention curriculum will be developed through a multi-stage process:

**Stage 1: Elicitation Study (December 2020)**

- Conduct an in-depth interview with 25 students (13 from the intervention and 12 from the control school)
- Explored salient beliefs about alcohol use
- Identified culturally relevant barriers and facilitators
- Informed content adaptation for local context

**Stage 2: Literature Review**

- Reviewing existing peer-led alcohol prevention programmes
- Examining TPB-based intervention studies
- Identifies evidence-based strategies and activities

**Stage 3: Expert Review**

- The curriculum will be reviewed by three public health experts.
- The curriculum will be reviewed by two school health educators
- The curriculum will be reviewed by two youth counsellors
- Modifications will be made based on feedback

**Stage 4: Pilot Testing (January 2021)**

- A pilot test will be conducted with 60 students (30 per school) not included in the main study
- Assessed comprehension, acceptability, and feasibility
- Refined activities and materials based on feedback

## *Peer Educator Selection and Training*

Peer educators will be selected based on demonstrated leadership qualities, effective communication skills, positive academic standing, and voluntary participation. A total of fifty students will be recruited and will undergo a comprehensive two-day intensive training programme prior to intervention delivery. The training content will include substance-related health and social consequences, principles of the Theory of Planned Behavior, behavior change communication strategies, facilitation techniques, ethical conduct, confidentiality, and interactive teaching methodologies.

The training will incorporate experiential components such as role-plays, rehearsal sessions, and scenario-based simulations to enhance competence and confidence. Two peer educators will be assigned to each intervention group to promote shared leadership, enhance session dynamics, and maintain implementation fidelity.

**Peer Educator Responsibilities:** Co-facilitate sessions with research team support; lead small group discussions; model positive behaviors; provide peer support; assist with activity implementation; and maintain confidentiality.

## *Intervention Modules Summary*

| Module | TPB Constructs Targeted | Key Activities | Duration |
|---|---|---|---|
| Module 1 | Knowledge & Risk Perception | Interactive presentation, small-group reflection, feedback | 60 min |
| Module 2 | Attitudes & Coping Skills | Structured debate, relaxation training, feedback | 60 min |
| Module 3 | Subjective Norms & Decision-Making | Guided discussion, decision-making practice, feedback | 60 min |
| Module 4 | Perceived Behavioral Control & Self-Efficacy | Goal-setting, role-play refusal skills, feedback | 60 min |

**Detailed Session Content**

**Module 1: Knowledge and Risk Perception (60 minutes)**

| Activity | Duration | Description |
|---|---|---|
| Interactive Presentation | 35 min | Health, psychological, and social consequences of alcohol; correcting myths |
| Small-Group Discussion | 20 min | Relate risks to real-life scenarios and share personal reflections |
| Feedback | 5 min | Complete session evaluation forms |

**Objectives:** Increase knowledge of alcohol-related harms, correct myths about alcohol use, and understand short-term and long-term consequences.

**Expected Outcomes:** Understanding immediate and long-term health risks, recognition of harmful alcohol constituents, evaluation of benefits of abstinence, and strengthened intentions to remain alcohol-free.

**Module 2: Attitudes and Coping Skills (60 minutes)**

**Activity 1: Structured Debate (45 minutes)**

- Topic: "Is using alcohol good or bad for someone your age?"
- Procedure: Group assignment (5 min), argument development (15 min), debate preparation (5 min), debate session (10 min), reflection (10 min)

**Activity 2: Relaxation Techniques (15 minutes)**

- Relaxation Exercise (5 min): Guided progressive muscle relaxation
- Deep Breathing (5 min): 4-count inhale, 8-count exhale (4-5 cycles)
- Homework: Practice daily

**Activity 3: Feedback (5 minutes)**

**Objectives:** Challenge positive attitudes toward alcohol, develop negative attitudes toward harmful use, and understand personal values and goals.

**Expected Outcomes:** Enhanced negative attitudes toward alcohol, recognition of alcohol-free identity benefits, and acquisition of anxiety management techniques.

**Module 3: Subjective Norms and Decision-Making (60 minutes)**

**Activity 1: Guided Discussion (40 minutes)**

Discussion prompts regarding significant others (parents, other family members, close friends, teachers):

- Would they approve/disapprove of your alcohol use?
- How would they influence you?

- How would you influence them?
- What positive things do you do for family/friends/teachers?
- What positive things do they do for you?
- What actions have you taken to help others reduce alcohol use?

**Activity 2: Decision-Making Practice (15 minutes)**

Based on Botvin (1980) 5-step model [27] :

1. Clarify the decision: What is the problem you are facing?
2. Consider possible choices: What options do you have?
3. Gather information: What else do you need to know?
4. Evaluate consequences: Advantages and disadvantages of each option?
5. Make and follow through: Which choice will you make?

**Activity 3: Feedback (5 minutes)**

**Objectives:** Correct misperceptions about peer alcohol use, develop skills to resist peer pressure, and identify positive social influences.

**Expected Outcomes:** Recognition of social influences on alcohol decisions, enhanced problem-solving and decision-making skills, and development of coping strategies for peer/family pressure.

**Module 4: Perceived Behavioral Control and Self-Efficacy (60 minutes)**

**Activity 1: Goal-Setting and Barrier Analysis (30 minutes)**

Students identify goals, barriers, and strategies for:

- Own alcohol use
- Friends' alcohol use
- Parents' alcohol use

Guiding Questions:

- What have you done to control alcohol use in your life or support others?
- What are your personal goals for avoiding alcohol?
- What barriers might make it difficult to achieve these goals?
- How could you overcome these barriers?

**Activity 2: Refusal Skills Role-Play (25 minutes)**

Part A: Identifying Peer Pressure Scenarios (10 minutes)

- Students share real or imagined pressure situations
- The facilitator lists scenarios on board
- Group discusses social dynamics and challenges

Part B: Role-Play Practice (15 minutes)

- Students practice assertive refusal skills, including a clear and confident "no", congruent body language and tone, offering alternative activities, and situation exit strategies

**Activity 3: Feedback (5 minutes)**

**Objectives:** Build self-efficacy to refuse alcohol, develop action plans for high-risk situations, and make a public commitment to avoid alcohol.

**Expected Outcomes:** Development of practical resistance skills, increased confidence in handling peer pressure, clear personal goals for alcohol-free living, and strategies for overcoming barriers.

**Intervention Fidelity Measures**

To ensure intervention fidelity, the following measures will be implemented:

- A facilitator manual with detailed step-by-step instructions for each session will be developed and provided to all peer educators
- Standardized materials, including identical presentations and activity guides, will be used across all intervention groups
- Research team members will be present at all sessions to observe and document implementation
- Ongoing supervision of peer educators will be conducted throughout the intervention period
- Fidelity checklists will be completed after each session to document adherence to the planned curriculum
- Debriefing meetings with regular feedback sessions will be held to address challenges and reinforce quality

## *Control Group Conditions*

Control group students will continue with their regular school activities and receive standard health education as part of the existing curriculum, which covers general topics such as hygiene, nutrition, and disease prevention but does not specifically address alcohol use prevention or incorporate Theory of Planned Behavior principles. No alcohol prevention sessions will be provided to control participants during the study period.

To ensure ethical equity, control group students will be offered the complete intervention materials and a summary session after study completion.

## *PLANS FOR OUTCOME ASSESSMENT AND DATA COLLECTION/ANALYSIS*

**Outcome Measures**

**Primary Outcomes**

| Outcome | Definition | Measurement | Scoring | Reliability/Validity |
|---|---|---|---|---|
| Behavioral Intention | Motivation or plan to drink alcohol within the next three months | 3 items, 7-point Likert scale (very unlikely to very likely) [15, 28] | Summed score 3-21; higher = stronger intention | Internal consistency will be considered acceptable if Cronbach's $\alpha \geq 0.70$. Test-retest reliability will be considered acceptable if intra-class correlation coefficient (ICC) $\geq$ 0.75. |
| Current Alcohol Use | Any alcohol consumption in the past 30 days | Single item: "In the past 30 days, on how many days did you have at least one drink of alcohol?" [29, 30] | Dichotomous (yes/no) | Test-retest reliability will be assessed using kappa statistic, with $\kappa \geq 0.60$ considered acceptable. |

***Secondary Outcomes***

| Outcome | Definition | Measurement | Scoring | Reliability/Validity |
|---|---|---|---|---|
| Alcohol-related Knowledge | Understanding of alcohol-related harms and myths | 23 yes/no/don't know items adapted from Eze et al. [31] | Summed score 0-23; higher = more knowledge | Internal consistency (Cronbach's $\alpha \geq 0.70$) and test-retest reliability (ICC $\geq$ 0.75) will be assessed. Content validity will be established through expert review. |
| Attitude (Direct) | Overall evaluation of alcohol consumption | 4 semantic differential items [15,28] | Summed score 4-28; higher = more favorable | Cronbach's $\alpha \geq 0.70$ will indicate acceptable internal consistency. ICC $\geq$ 0.75 will indicate acceptable test-retest reliability. |
| Attitude (Indirect) | Belief-based evaluation | 12 belief items × 12 outcome evaluation items [28] | Range: -84 to +84 | Cronbach's $\alpha \geq 0.70$ will be considered acceptable. Construct validity will be assessed through confirmatory factor analysis. |
| Subjective Norms (Direct) | Perceived social pressure | 4 items, 7-point Likert scale [15,28] | Summed score 4-28; higher = more approval | Acceptable reliability will be defined as Cronbach's $\alpha \geq 0.70$ and ICC $\geq 0.75$. |

| Outcome | Definition | Measurement | Scoring | Reliability/Validity |
|---|---|---|---|---|
| Subjective Norms (Indirect) | Normative beliefs × motivation to comply | 6 normative belief items × 6 motivation items [28] | Range: -84 to +84 | Cronbach's α ≥ 0.70 will indicate acceptable internal consistency. |
| Perceived Behavioral Control (Direct) | Perceived ability to resist alcohol | 4 items, 7-point Likert scale [15, 28] | Summed score 4-28; higher = more control | Cronbach's α ≥ 0.70 and ICC ≥ 0.75 will be considered acceptable. |
| Perceived Behavioral Control (Indirect) | Control beliefs × perceived power | 7 control belief items × 7 power items [28] | Range: -105 to +105 | Internal consistency will be assessed with Cronbach's α, with α ≥ 0.70 considered acceptable. |

**Covariates**

| Domain | Variables |
|---|---|
| Demographics | Age (years, categorized as 15-19, 20-24), Sex (male, female), Grade level (9-10 lower secondary, 11-12 upper secondary), Religion (Orthodox, Muslim, Protestant, Other), Ethnicity (Amhara, Oromo, Tigray, Other) |
| Socioeconomic | Father's education (no formal, primary, secondary, or higher); Mother's education (no formal, primary, secondary, or higher) |
| Family Factors | Family discussion about alcohol (yes/no), parental alcohol use (yes/no) |
| Peer Factors | Peer alcohol use (yes/no) |

## Data Collection Procedures

## Questionnaire Development

The self-administered questionnaire will be developed through adaptation of standardized TPB questionnaires [28], review of relevant literature [16, 17, 31], incorporation of findings from elicitation study, translation and back-translation (English → Amharic → English), and expert review by public health specialists and language experts.

## Pre-testing and Validation

The questionnaire will be pre-tested with 60 students (30 per school) not included in the main study to assess the following:

- **Internal consistency:** Cronbach's $\alpha$ will be calculated for each multi-item scale, with $\alpha \geq 0.70$ considered acceptable
- **Test-retest reliability:** Intra-class correlation coefficients (ICC) will be calculated by administering the questionnaire to the same students two weeks apart, with ICC $\geq 0.75$ considered acceptable; for the single-item current alcohol use measure, kappa statistic ($\kappa \geq 0.60$) will be used
- **Content validity:** Expert review by public health specialists and language experts will ensure items adequately cover the construct domains
- **Face validity:** Pilot participants will be asked whether items are clear, relevant, and acceptable

## Data Collection Schedule

| Time Point | Activity | Instruments |
| --- | --- | --- |
| Baseline (February 1-7, 2021) | Self-administered questionnaire | TPB questionnaire, knowledge items, demographic form |
| Intervention (February 8 - March 7, 2021) | 4 weekly sessions | Session logs, fidelity checklists |
| Endline (June 7-15, 2021) | Self-administered questionnaire | Same as baseline |

## *Data Collection Procedures*

**Baseline Data Collection (Scheduled for February 1-7, 2021):**

- Research assistants will be trained for 2 days on data collection procedures prior to baseline assessment
- Questionnaires will be distributed in classrooms during regular school hours
- Standardized instructions will be read aloud by research assistants to ensure consistency
- Students will complete questionnaires independently, with an expected completion time of 45-60 minutes
- Research assistants will remain in the classroom to answer questions and provide clarification as needed
- Completed questionnaires will be collected immediately to minimize data loss

**Endline Data Collection (Scheduled for June 7-15, 2021):**

- The same procedures as the baseline will be followed
- Data collection will be conducted three months post-intervention
- The same validated questionnaires will be used to ensure comparability
- Different research assistants will be deployed for endline data collection to maintain blinding and reduce measurement bias

## *Quality Control Measures*

| Measure | Description |
|---|---|
| Standardized training | All data collectors will receive 2-day training |
| Field manual | Detailed manual provided to all data collectors |
| Supervision | Regular supervision during data collection |
| Daily checks | Completed questionnaires checked daily for completeness |
| Immediate feedback | Corrective action taken when needed |
| Double data entry | Two independent operators to minimize errors |

## *Data Management and Statistical Analysis*

**Data Entry and Management**

| Activity | Description |
|---|---|
| Data Entry | Data will be double-entered using EpiData 3.1 software, with range and consistency checks built into entry screens two independent data entry operators; entries will be compared and discrepancies resolved by referring to original questionnaires |
| Data Cleaning | Frequency distributions will be checked for out-of-range values; consistency checks across related variables; missing data patterns will be examined; outliers will be identified and verified |
| Data Storage | De-identified data stored on password-protected computers; backup copies will be maintained on secure cloud storage; paper questionnaires will be stored in locked cabinets; access limited to research team members |
| Data Security | Participant identifiers will be removed from the analysis dataset; linking codes will be kept separately from data; secure transfer protocols will be used for data sharing |

## *Statistical Analysis Plan*

***Descriptive Analysis***

Descriptive statistics will be computed to summarize the baseline characteristics of study participants:

- **Frequencies and percentages** will be calculated for all categorical variables (e.g., sex, grade level, religion, parental education, family discussion about alcohol, and peer alcohol use).
- **Means and standard deviations** will be computed for continuous variables that are normally distributed (e.g., age, knowledge scores, and TPB construct scores).
- **Medians and interquartile ranges** will be computed for continuous variables that are not normally distributed
- **Tables and figures** will be generated to summarize baseline characteristics and to facilitate comparison between intervention and control groups prior to the intervention

Normality of continuous variables will be assessed using Shapiro-Wilk tests and visual inspection of Q-Q plots prior to selecting appropriate descriptive statistics.

## ***Baseline Comparability:***

| Variable Type | Test |
|---|---|
| Categorical variables | Chi-square tests |
| Continuous variables (normal distribution) | Independent t-tests |
| Continuous variables (non-normal distribution) | Mann-Whitney U tests |

Variables with $p < 0.05$ will be considered unbalanced and adjusted in multivariable analyses.

**Within-Group Changes:**

| Outcome Type | Test | Effect Size |
|---|---|---|
| Continuous | Paired t-tests | Cohen's d |
| Dichotomous | McNemar's tests | Odds ratios |

**Between-Group Comparisons:**

| Outcome Type | Test | Effect Size |
|---|---|---|
| Continuous | Independent t-tests | Cohen's d with 95% CI |
| Dichotomous | Chi-square tests | Odds ratios with 95% CI |

**Generalized Estimating Equations (GEE)** will be used to account for clustering at the class level:

| Component | Specification |
|---|---|
| Distribution family | Normal for continuous outcomes; binomial for dichotomous outcomes |
| Link function | Identity for continuous; logit for dichotomous |
| Correlation structure | Exchangeable (assumes equal correlation within clusters) |

| Component | Specification |
|---|---|
| Clustering unit | Class level |

**Adjusted Variables:**

- Baseline value of outcome variable
- Father's education
- Mother's education
- Family discussion about alcohol
- Baseline alcohol use status

**Model Fit Assessment:**

- Quasi-likelihood under Independence Model Criterion (QIC) for model comparison
- Examination of residuals
- Sensitivity analysis using different correlation structures

**Software**

| Purpose | Software |
|---|---|
| Data entry | EpiData 3.1 |
| Statistical analysis | SPSS version 25 |

## *Handling of Missing Data*

**Missing Data Assessment:**

- The proportion of missing data will be calculated for each variable

**Handling Strategy:**

- **Complete-case analysis** will be conducted for the primary analysis, as the anticipated proportion of missing data is low (< 2%).
- **Multiple imputation** using chained equations will be performed if missing data exceed 5% to reduce potential bias and preserve statistical power

## *References*


1. World Health Organization. Global status report on alcohol and health 2018. Geneva: World Health Organization; 2018.
2. World Health Organization. Global Health Observatory (GHO) data: Alcohol consumption among adolescents 15–19 years. Geneva: World Health Organization; 2018.
3. Rehm J, Shield KD. Global burden of disease and economic cost attributable to alcohol use and alcohol-use disorders. Lancet. 2013;381(9872):988–1000. doi: 10.1016/S0140-6736(13)61019-6
4. Squeglia LM, Jacobus J, Tapert SF. The influence of substance use on adolescent brain development. Clinical EEG and Neuroscience. 2009;40(1):31–38. doi: 10.1177/155005940904000110
5. Cairns KE, Yap MB, Pilkington PD, Jorm AF. The risk of adolescent suicide across patterns of drug use: a nationally representative study of high school students in the United States. Journal of Adolescent Health. 2014;55(3):432–437. doi: 10.1016/j.jadohealth.2014.02.004
6. Stueve A, O'Donnell LN. Early alcohol initiation and subsequent sexual and alcohol risk behaviors among urban youths. American Journal of Public Health. 2005;95(5):887–893. doi: 10.2105/AJPH.2003.026567
7. Latvala A, Rose RJ, Pulkkinen L, Dick DM, Kaprio J. Drinking, smoking, and educational achievement: genetic and environmental influences on young adult outcomes. Twin Research and Human Genetics. 2014;17(1):1–13. doi: 10.1017/thg.2013.82
8. Grant BF, Dawson DA. Age at onset of alcohol use and its association with DSM-IV alcohol abuse and dependence: results from the National Longitudinal Alcohol Epidemiologic Survey. Journal of Substance Abuse. 1997; 9:103–110. doi: 10.1016/s0899-3289(97)90009-2
9. Central Statistical Agency (CSA) [Ethiopia] and ICF. Ethiopia Demographic and Health Survey 2016. Addis Ababa, Ethiopia, and Rockville, Maryland, USA: CSA and ICF; 2017.
10. Ali T, Worku T. Current alcohol consumption and associated factors among school adolescents and youths in Ethiopia: a systematic review and meta-analysis. SAGE Open Medicine. 2020; 8:2050312120974154. doi: 10.1177/2050312120974154

11. Wondimu H. The prevalence and determinant factors of alcohol abuse among preparatory school students in Gondar, Ethiopia. PLoS ONE. 2023;18(4):e0277966. doi: 10.1371/journal.pone.0277966
12. Shibiru T, Mengistu D, Egata G, Ayele G. Prevalence and factors associated with alcohol consumption among secondary school students in Nekemte town, Ethiopia. BMC Public Health. 2023;23(1):123. doi: 10.1186/s12889-023-16088-9
13. Tulu SK, Fekadu A, Girma E, Adugna A. Prevalence and associated factors of alcohol use among high school students in Addis Ababa, Ethiopia. Substance Abuse Treatment, Prevention, and Policy. 2018;13(1):43. doi: 10.1186/s13011-018-0179-3
14. Tesfaye G, Derese A, Hambisa MT. Substance use and associated factors among university students in Ethiopia: a cross-sectional study. Journal of Addiction. 2014; 2014:969837. doi: 10.1155/2014/969837
15. Ajzen I. The theory of planned behavior. Organizational Behavior and Human Decision Processes. 1991;50(2):179–211. doi: 10.1016/0749-5978(91)90020-T
16. Cooke R, Dahdah M, Norman P, French DP. How well does the theory of planned behaviour predict alcohol consumption? A systematic review and meta-analysis. Health Psychology Review. 2016;10(2):148–167. doi: 10.1080/17437199.2014.947547
17. McEachan RRC, Conner M, Taylor NJ, Lawton RJ. Prospective prediction of health-related behaviours with the theory of planned behaviour: a meta-analysis. Health Psychology Review. 2011;5(2):97–144. doi: 10.1080/17437199.2010.521684
18. Topa G, Moriano JA. Theory of planned behavior and smoking: meta-analysis and SEM model. Substance Abuse and Rehabilitation. 2010;1:23–33. doi: 10.2147/SAR.S15168
19. Marcoux BC, Shope JT. Application of the theory of planned behavior to adolescent use and misuse of alcohol. Health Education Research. 1997;12(3):323–331. doi: 10.1093/her/12.3.323
20. Collins SE, Carey KB. The theory of planned behavior as a model of heavy episodic drinking among college students. Psychology of Addictive Behaviors. 2007;21(4):498–507. doi: 10.1037/0893-164X.21.4.498
21. Sheppard CS, Ellis JB, Patton R. Peer-led interventions for reducing alcohol, tobacco and drug use among adolescents: a systematic review. International Journal of Adolescent Medicine and Health. 2017;29(5). doi: 10.1515/ijamh-2015-0107
22. MacArthur GJ, Harrison S, Caldwell DM, Hickman M, Campbell R. Peer-led interventions to prevent tobacco, alcohol and/or drug use among young people aged

11–21 years: a systematic review and meta-analysis. Addiction. 2016;111(3):391–407. doi: 10.1111/add.13224

23. Gebremariam TB, Mruts KB, Neway TK. Substance use and associated factors among Debre Berhan University students, Central Ethiopia. Substance Abuse Treatment, Prevention, and Policy. 2018;13(1):13. doi: 10.1186/s13011-018-0150-3
24. Reda AA, Moges A, Wondmagegn BY, Biadgilign S. Alcohol drinking patterns among high school students in Ethiopia: a cross-sectional study. BMC Public Health. 2012; 12:213. doi: 10.1186/1471-2458-12-213
25. Donner A, Klar N. Design and analysis of cluster randomization trials in health research. London: Arnold; 2000.
26. Murray DM. Design and analysis of group-randomized trials. New York: Oxford University Press; 1998.
27. Botvin GJ. Life skills training: teacher's manual. New York: Smithfield Press; 1980.
28. Francis JJ, Eccles MP, Johnston M, Walker A, Grimshaw JM, Foy R, et al. Constructing questionnaires based on the theory of planned behaviour: A manual for health services researchers. Newcastle upon Tyne: Centre for Health Services Research, University of Newcastle; 2004.
29. National Institute on Drug Abuse. National Survey on Drug Use and Health (NSDUH): summary of methodological studies 1971–2014. Bethesda: National Institutes of Health; 2014.
30. World Health Organization. WHO STEPS surveillance manual: the WHO STEPwise approach to chronic disease risk factor surveillance? Geneva: World Health Organization; 2017.
31. Eze NM, Njoku HA, Eseadi C, Akubue BN, Ezeanwu AB, Ugwu UC, et al. Alcohol consumption and awareness of its effects on health among secondary school students in Nigeria. Substance Use and Misuse. 2017;52(12):1636–1643. doi: 10.1080/10826084.2017.1349798

**Protocol Prepared By:** Yitbarek Wasihun, (Principal Investigator)

**Date:** January 15, 2021

**Version:** 1.0
